# A Model to Predict Psychological- and Health-Related Adjustment in Men with Prostate Cancer: The Role of Post Traumatic Growth, Physical Post Traumatic Growth, Resilience and Mindfulness

**DOI:** 10.3389/fpsyg.2018.00136

**Published:** 2018-02-15

**Authors:** Deirdre M. J. Walsh, Todd G. Morrison, Ronan J. Conway, Eamonn Rogers, Francis J. Sullivan, AnnMarie Groarke

**Affiliations:** ^1^School of Psychology, National University of Ireland Galway, Galway, Ireland; ^2^Department of Psychology, University of Saskatchewan, Saskatoon, SK, Canada; ^3^School of Psychology, University College Dublin, Dublin, Ireland; ^4^University Hospital Galway, Galway, Ireland; ^5^Prostate Cancer Institute, National University of Ireland Galway, Galway, Ireland

**Keywords:** post traumatic growth, physical post traumatic growth, scale development, corporeal post traumatic growth, prostate cancer, resilience (psychology)

## Abstract

**Background:** Post traumatic growth (PTG) can be defined as positive change following a traumatic event. The current conceptualization of PTG encompasses five main dimensions, however, there is no dimension which accounts for the distinct effect of a physical trauma on PTG. The purpose of the present research was to test the role of PTG, physical post traumatic growth (PPTG), resilience and mindfulness in predicting psychological and health related adjustment.

**Method:** Ethical approval was obtained from relevant institutional ethics committees. Participants (*N* = 241), who were at least 1 year post prostate cancer treatment, were invited to complete a battery of questionnaires either through an online survey or a paper and pencil package received in the post The sample ranged in age from 44 to 88 years (*M* = 64.02, *SD* = 7.76). Data were analysis using confirmatory factor analysis and structural equation modeling.

**Results:** The physical post traumatic growth inventory (P-PTGI) was used to evaluate the role of PPTG in predicting adjustment using structural equation modeling. P-PTGI predicted lower distress and improvement of quality of life, whereas conversely, the traditional PTG measure was linked with poor adjustment. The relationship between resilience and adjustment was found to be mediated by P-PTGI.

**Conclusion:** Findings suggest the central role of PTG in the prostate cancer survivorship experience is enhanced by the inclusion of PPTG. Adjusting to a physical trauma such as illness (internal transgressor) is unlike a trauma with an external transgressor as the physical trauma creates an entirely different framework for adjustment. The current study demonstrates the impact of PPTG on adjustment. This significantly adds to the theory of the development of PTG by highlighting the interplay of resilience with PTG, PPTG, and adjustment.

## Introduction

Post traumatic growth (PTG) is defined as a collection of positive changes following a traumatic event. In the development of PTG, a traumatic event acts as a catalyst for the individual to re-evaluate his or her worldview. This can often result in distress but also various forms of positive growth (Tedeschi and Calhoun, 1996). Quantitative research in the area of PTG has revealed that, after a traumatic event, life changes often occur in five domains: *personal strength*; *social relationships*; *appreciation for life*; *identification of new possibilities*; and *changes to spirituality* (Tedeschi and Calhoun, 1996, 2004). However, past research has not fully considered the distinct role that physical- and health-related traumas, such as cancer, may play in PTG (Barskova and Oesterreich, 2009). The experience of physical trauma has elements unique from other traumas due to its internalized nature and direct impact on the body. Physical post traumatic growth (PPTG) or corporeal PTG can be defined as an aspect of growth directly resulting from experiencing a physical trauma. Physical trauma can facilitate a ‘reconnection to the body’ with specific positive outcomes including (1) enhanced appreciation for the body, (2) increased care toward the body (listening to the body; treating it better) and (3) increased health behavior changes (Hefferon, 2012). This dimension of growth is distinct from the five dimensions currently posited by Tedeschi and Calhoun (1996) and it is important for researchers to further investigate the broad range of physical and psychological benefits found in survivors exhibiting PTG including those related to the physical self (Urcuyo et al., 2005; Stanton et al., 2006).

Previous attempts have been made to extend the main model of PTG (Tedeschi and Calhoun, 2004). Sabiston et al. (2007), for example, articulated the first model of PTG which included other factors than those traditionally defined. They presented a revised model of positive growth which included a focus on physical activity and feelings of personal control, in addition to changes in perceptions of the physical self (e.g., strength and fitness). This is an important avenue to explore as the corporeal nature of physical trauma may have wide implications for adjustment (Hefferon et al., 2009, 2010; Hefferon, 2012).

Of interest in the current study are survivor groups of men with prostate cancer who may be experiencing ongoing side effects following treatment. Apart from skin cancer, prostate cancer is the most common cancer in men (Vanagas et al., 2013).

Due to the high survival rates and reports of men living with many long-term issues impacting survivors, it is essential to examine psychological adjustment within the cancer survivorship journey (Curtis et al., 2014b). Sanda et al. (2008) found that all prostate cancer treatment has a unique trajectory in terms of changes in quality of life impacting on urinary, sexual, bowel, and hormonal functioning. They highlighted the importance of physical side effects in the experience of prostate cancer survivorship including quality of life and well-being. Thus, physical trauma and subsequent related constructs (e.g., body awareness) should be included in future models of PTG.

To date, empirical evidence regarding the predictors and consequences of PTG is inconclusive (Groarke et al., 2016). The proposed model seeks to examine the role of PTG and PPTG in men with prostate cancer on body awareness, distress and quality of life. To the researchers’ knowledge, the current study is the first one to delineate a model of PTG that includes PPTG. As such, it is important to consider how variables such as resilience, distress and quality of life, which are commonly assessed with PTG, may interact with the addition of PPTG. It is also critical to incorporate other variables which may be specific to physical trauma such as mindfulness and body awareness.

### Cognitive Processing within PTG and PPTG

#### Resilience, PTG, and physical PTG

Post traumatic growth has been viewed as a form of cognitive adaptation in response to a cancer diagnosis that can give meaning and act as a buffer against distress in breast cancer survivors (Helgeson et al., 2006). Inconsistent findings between PTG and adjustment may be influenced by an individual’s resilience and cognitive processing (Stockton et al., 2011). Resilience can be defined as an ability to recalibrate your personal worldview in relation to cognitions, beliefs, and behaviors. This, in turn, can facilitate flexibility which can aid some people in adapting following a traumatic experience (Walsh, 1998). There have been conflicting research findings with regard to the relationship between PTG and resilience (Bonanno et al., 2004). For example, Duan et al. (2015) found that resilience was positively associated with PTG, while other studies find high levels of resilience associated with the lowest PTG scores (Levine et al., 2009). Tedeschi and Calhoun (1996) have posited that those who are most resilient may experience PTG to a lesser extent as the traumatic experience may be less challenging to them and thus may not act as a catalyst for meaning-making (Bonanno et al., 2004) and the extensive cognitive processing associated with growth. Given that cognitive processing is the most essential element in the model of PTG, it has been argued that, perhaps, resilience may not facilitate PTG (Westphal and Bonanno, 2007). We need, however, more empirical investigations of how resilience relates to an individual’s perception of positive growth. Within the current study, we will explore whether resilience is a predictor of well-being and distress. This model will also test whether PTG and PPTG mediate the relationship between resilience and outcome variables (i.e., distress, quality of life, and body awareness).

#### Mindfulness, PTG, and PPTG

Mindfulness can be considered an aspect of cognitive processing due to its central factors of acceptance and awareness. This is important given the evidenced centrality of cognitive processing in the growth process (Nightingale et al., 2010; Stockton et al., 2011; Joseph et al., 2012). In the current model, mindfulness will be assessed as a direct pathway to adjustment indices. Some elements of mindfulness are thought of as useful buffers which can be used in the face of issues of distress (Kabat-Zinn, 1990; Hayes and Feldman, 2004; Keng et al., 2011). A meta-analysis of randomized controlled trials has also shown an average medium-sized effect of mindfulness (*d* = 0.54) on a composite score of psychological well-being (Grossman et al., 2004). Therefore, in the current model, it is proposed that mindfulness is related to both emotional outcomes and health-related change due to the central cognitive component present which influences PTG. It is hypothesized that higher levels of mindfulness, encapsulating awareness and acceptance, will relate to lower distress in line with previous research. The expectation is that those higher in mindfulness will experience lower distress and greater quality of life and body awareness. Thus, it is hypothesized that those with higher levels of mindfulness will experience greater levels of quality of life following prostate cancer.

The way in which mindfulness is currently defined (namely, ‘orienting to one’s internal and external experiences’: Gilbert and Waltz, 2010, p. 227) inextricably links the two constructs of mindfulness and body awareness. This may indicate that higher levels of mindfulness predict greater levels of body awareness. Mindfulness has also been shown to facilitate greater psychosomatic awareness in various clinical populations (Keng et al., 2011).

### Adjustment within PTG and PPTG

#### Distress, PTG, and PPTG

Nightingale et al. (2010) found links between psychological distress and PTG. Previous meta-analyses demonstrate that psychological distress in men with prostate cancer is a serious issue, with Watts et al. (2014) reporting a high prevalence of depression and anxiety in men with prostate cancer prior to and during treatment (e.g., pre-treatment depression 17.2% and anxiety 27.4%). Importantly, previous investigations highlight how after physical trauma patients can experience positive outcomes while, in parallel, experience distress. Morrill et al. (2008) reported that PTG moderated associations between post-traumatic stress and distress quality of life. They showed that PTG may work to counter certain negative aspects of post-traumatic stress in a sample of breast cancer survivors by acting as a positive buffer alleviating some distress and, perhaps, contributing to well-being. This then could have implications for long term adjustment. Indeed, the importance of distress within the adjustment process is becoming recognized as the International Psycho-oncology Society now recommends that distress be assessed as an additional vital sign within a patient consultation (Watson and Jacobsen, 2012).

Unfortunately, previous research has not yet been able to identify a clear and consistent relationship between traditional PTG and distress. In a previous meta-analysis, PTG was correlated with lower levels of depression (Helgeson et al., 2006), Groarke et al. (2016) found that high levels of cancer-specific distress were correlated with positive growth. These findings support how dealing with physical trauma can initiate PTG and potentially impact longer-term adjustment. Morris and Shakespeare-Finch (2011) conducted analyses using data obtained from 313 participants diagnosed with a variety of cancers. Their model provided support that following cancer diagnosis, an individual’s survivorship journey is *simultaneously* influenced by both positive and negative experiences and that either outcome may be more prevalent or may even occur concurrently. These results highlight the importance of investigating both growth and distress arising from the same trauma (Curtis et al., 2014a; Leal et al., 2014) The current study seeks to further explore the relationship between PTG and distress (e.g., Helgeson et al., 2006; Nightingale et al., 2010) and to explore how the new concept of PPTG links with PTG and distress.

#### Quality of Life

The relationship between PTG and quality of life following physical trauma is still unclear. Thornton and Perez (2006) found that PTG was largely unrelated to quality of life outcomes in prostate cancer survivors. This adds to the growing body of inconsistent findings as some investigators have reported that PTG is largely unrelated to quality of life (Fromm et al., 1996; Cordova et al., 2001; Sears et al., 2003) and others have found that it is inversely related (Tomich and Helgeson, 2004).

Typically, studies use the post traumatic growth inventory (PTGI) which measures psychosocial areas of growth but not physical domains (Bellizzi et al., 2010). In addition, measures of quality of life may not adequately capture the ‘existential flavor’ (Thornton and Perez, 2006, p. 293) of the benefits reported by cancer survivors. It is possible that inconsistencies in the literature stem from an individual reporting higher traditional PTG and lower quality of life scores, because higher traditional PTG may not capture the corporeal nature of illness and physical trauma (Thornton and Perez, 2006) and, therefore, does not predict quality of life. In the current study, higher *physical* PTG is hypothesized to predict higher quality of life.

#### Body Awareness

Physical trauma can facilitate a renewed sense of the physical self where individuals feel more connected to their bodies and its physical functioning (Frank, 1995; Hefferon et al., 2009). This has been conceptualized through key themes emerging from previous literature (1) enhanced appreciation for the body, (2) looking after the body better, and (3) greater levels of healthy lifestyle behaviors (i.e., teachable moments: see Demark-Wahnefried et al., 2000). It is hypothesized that those with higher levels of PTG and physical PTG will exhibit higher levels of body awareness.

The primary objective of the current study is to explore the role of PTG, PPTG, resilience and mindfulness in the psychological and health-related adjustment in men with prostate cancer. A summary of study hypotheses and exploratory (non-directional) hypotheses are below:

(1) PTG and PPTG mediate the relationship between resilience and outcome variables (i.e., distress, quality of life and body awareness).
(2) Higher levels of mindfulness will be associated with lower distress and greater levels of quality of life following prostate cancer.
(3) Higher PPTG will be associated with greater quality of life.
(4) Higher levels of PTG and PPTG will be correlated with higher levels of body awareness.
(5) The relationships among PTG, PPTG, and distressed will be explored.
(6) Whether resilience is a predictor of well-being and distress also will be investigated.

## Materials and Methods

### Procedure

Ethical approval was obtained from relevant institutional ethics committees. Participants, who were at least 1 year post prostate cancer treatment, were invited to complete a battery of questionnaires either through an online survey or a paper and pencil package received in the post. Final enrolment for this study was in Spring 2014 (enrolment period from October 2013 to April 2014).

### Participants

The total number of participants was 241. When the online and paper and pencil samples were compared, a statistically significant difference in age was found, *t*(239) = -3.33, *p* < 0.01, Cohen’s *d* = 0.44, with the online sample being younger (*M*_age_ = 62.72 years, *SD* = 7.58) than the postal sample (*M*_age_ = 66.05 years, *SD* = 7.60). No difference was found between the groups in terms of ‘time since diagnosis’ (*p* > 0.05). In addition, no difference was found between the three ‘time since diagnosis’ groups [1–2 years (*n* = 103, 42.7%), 3–4 years (*n* = 64, 26.6%), 5–10 years (*n* = 74, 30.7%)] on PTG and physical PTG (*p*s > 0.05).

The sample ranged in age from 44 to 88 years (*M* = 64.02, *SD* = 7.76). A majority of the sample were from either North America (46.90%; *n* = 113) or Europe (47.7%; *n* = 115). Five treatment groups were represented: surgery only (22.0%, *n* = 53), radiotherapy only (30.7%, *n* = 74), hormone therapy only (3.3%, *n* = 8), combination (33.2%, *n* = 80) and other (5.4%, *n* = 13). In addition, some participants had not opted for treatment, choosing ‘active surveillance’ (5.0%, *n* = 12).

### Materials

#### Post Traumatic Growth Inventory (PTGI; Tedeschi and Calhoun, 1996)

The PTGI is a 21-item scale that provides a measure of positive change following trauma. The scale contains five dimensions: relating to others, new possibilities, personal strength, appreciation of life and spiritual change (Tedeschi and Calhoun, 1996). Participants are asked to rate the level of positive change they perceive in their lives as a result of the trauma. Responses are coded on a six-point Likert scale ranging from 0 (not at all) to 5 (a very great deal) with higher scores indicating greater levels of positive life changes (Tedeschi and Calhoun, 1996).

Adequate reliability and validity have been demonstrated including estimates of internal consistency and test–re-test reliability, concurrent and discriminant validity (Tedeschi and Calhoun, 1996). Tedeschi and Calhoun (1996) also reported that scores on the PTGI were not significantly associated with social desirability (Crowne and Marlowe, 1960). In the current study, Cronbach’s alpha for the PTGI was 0.97 (95% CI = 0.96–0.97).

#### Physical Post Traumatic Growth Scale (P-PTGI; Walsh and Groarke, unpublished)

The P-PTGI scale consists of 20 items. These items aim to assess PTG following physical trauma. Respondents are asked to consider how they feel now after diagnosis and treatment on a six-point Likert scale ranging from -2 (greatly decreased) to 2 (greatly increased). The scale also includes options such as 0 (no change) and 3 (not applicable to me). The ‘not applicable to me’ option is to be coded as 99 (missing value) and mean scores should be calculated rather than total scores. Higher scores indicate greater PPTG. Reliability of the P-PTGI is excellent, with subscales displaying high internal reliability. To illustrate: reliability was found to be 0.92 (95% CI = 0.90–0.93; health awareness) and 0.91 (95% CI = 0.89–0.93; health autonomy), respectively, with a total scale reliability of 0.92 (95% CI = 0.90–0.93).

Construct validity of the P-PTGI was supported, with scores on the P-PTGI correlating positively with traditional PTG (total score and its subscales), and with mindfulness.

#### Connor–Davidson Resilience Scale (CD-RISC; Connor and Davidson, 2003)

The CD-RISC measures perceptions of stress and coping ability. Participants are asked to rate 10 items based on how they have felt in the past month on a five-point Likert scale ranging from 0 (not true at all) to 4 (true nearly all of the time) (Connor and Davidson, 2003). Higher total scores indicate greater coping ability (possible range is 0 to 40). Cronbach’s alpha for the current study was 0.93 for resilience (95% CI = 0.91–0.94). The CD-RISC has previously demonstrated adequate construct validity. Scores on the CD-RISC has previously been able to differentiate between groups reporting levels of psychiatric symptoms following childhood maltreatment (Campbell-Sills and Stein, 2007).

#### The Freiburg Mindfulness Inventory (FMI; Walach et al., 2006)

The FMI is a 14-item scale that provides a brief measure of mindfulness. Responses are coded on a four-point Likert scale from 1 (rarely) to 4 (almost always). Higher scores denote higher levels of mindfulness (possible range is 14–56). Construct validity has been tested, with scores on the FMI correlating positively with self-awareness and negatively with dissociation (Walach et al., 2006) as well as depression (Kohls et al., 2009). In the current study, scale score reliability was found to be excellent (alpha = 0.91, 95% CI = 0.89–0.93).

#### Private Body Consciousness Subscale (PBCS) of the Body Consciousness Questionnaire

This is a widely used measure of body awareness (e.g., Mehling et al., 2009) consisting of three dimensions (‘public body consciousness,’ ‘private body consciousness,’ and ‘body competence’). The PBCS is a 5-item subscale of the Body Consciousness Questionnaire (BCQ) that can be defined as a tendency to focus on internal body sensations, being aware of interoceptive feedback, and being sensitive to changes in the body and physical functioning (Miller et al., 1981). Each item on the scale ranged from 0 (extremely uncharacteristic) to 4 (extremely characteristic). Scores may range from 0 to 20, with higher scores denoting greater body consciousness (Miller et al., 1981). The instrument has been used with a variety of patient populations (Ferguson and Ahles, 1998) with similar scores reported across different groups and controls, supporting how the construct is independent from illness (i.e., chronic pain). For the current sample, Cronbach’s alpha for body awareness was 0.77 (95% CI = 0.72–0.81).

#### Patient-Oriented Prostate Utility Scale (PORPUS; Ritvo et al., 2005)

The PORPUS contains 10 questions: Five are general (pain, energy, emotional well-being, social well-being, and relationship with physician) and five are prostate cancer-specific (sexual function and desire, urinary frequency and incontinence, and bowel function) (Ritvo et al., 2005). Each question has a range of answers specifically related to the question (e.g., pain) on a Likert scale ranging from ‘no pain and no disturbing body sensations’ to ‘severe pain or disturbing sensations that limit many activities.’ Within the current study, lower scores on this quality of life measure denote greater quality of life.

For the current study, reliability analyses were conducted on two factors within the PORPUS. Factor 1 (physical symptoms) had a Cronbach’s alpha of 0.69 (95% CI = 0.62–0.75). Factor 2 (emotional well-being) had a Cronbach’s alpha of 0.63 (95% CI = 0.55–0.71). It should be noted that the latter value is below the threshold for satisfactory scale score reliability (Nunnally, 1978).

#### The Hospital and Anxiety Depression Scale (HADS; Zigmond and Snaith, 1983)

The HADS is a 14-item scale measure of distress (i.e., anxiety and depression). Responses are coded on a four-point Likert scale (e.g., 0 = not at all, 3 = most of the time; 0 = definitely as much, 3 = hardly at all) (Zigmond and Snaith, 1983). Higher scores denote greater anxiety or depression (possible range for each seven-item subscale is 0–21) (Zigmond and Snaith, 1983). Previous research has found the HADS to be reliable and valid (Zigmond and Snaith, 1983; Bjelland et al., 2002). In a review of all studies that employed the HADS, Cronbach’s alpha values were greater than 0.60 indicating adequate scale score reliability (Bjelland et al., 2002). In the current study, Cronbach’s alpha was 0.86 (95% CI = 0.84–0.89) for the total HADS, 0.82 (95% CI = 0.78–0.85) for the anxiety subscale, and 0.78 (95% CI = 0.74–0.82) for the depression subscale.

### Data Analysis

Statistical software packages were used for data analysis (i.e., IBM AMOS 23 and SPSS 22). The factor structure of each measure was determined using a combination of exploratory and confirmatory factor analyses (EFA and CFA). This method ensured that all variables were accurately measuring their assigned construct. For some scales (e.g., PORPUS Quality of Life measure and the FMI), the use of CFA was not appropriate as the dimensionality of the measure had not been determined previously.

Structural equation modeling (SEM) was then used to test the proposed model. SEM is a statistical means of examining proposed relationships among hypothetical latent constructs which are indicated by observed variables, allowing for the separation of the measurement and structural components of the model. Missing data levels were less than 5% and were treated using Expectation–Maximization (EM). The normality of the data was assessed, with skewness and kurtosis at acceptable levels (i.e., skewness < 3; kurtosis < 10; skew; Chou and Bentler, 1995; Weston and Gore, 2006; Kline, 2011).

Mediation analysis tests the effect of a variable (i.e., PPTG) that accounts for the relation between a predictor variable (e.g., resilience) and an outcome variable (e.g., quality of life; Baron and Kenny, 1986). Thus, in the current study, to test for mediation, Hayes’ (2013) method of testing mediation using AMOS 23 was conducted to assess whether PTG and PPTG mediated the relationship between resilience and each outcome (i.e., anxiety, depression, and quality of life).

### CFA Tests of Dimensionality

#### Post Traumatic Growth Inventory (PTGI; Tedeschi and Calhoun, 1996)

The PTGI measure illustrated a poor fit.; χ^2^(179) = 666.87, *p* < 0.001, *Q* = 3.73, CFI = 0.89, TLI = 0.87, RMSEA = 0.107 (95% CI 0.098–0.115), AIC = 770.87, SRMR = 0.05. Modification indices suggested a number of covariances such as items 1 and 2 (‘I changed my priorities about what is important in life’ and ‘I have a greater appreciation for the value of my own life’), items 20 and 21 (‘I learned a great deal about how wonderful people are’ and ‘I better accept needing others’), items 15 and 16 (‘I have more compassion for others’ and ‘I put more effort into my relationships’) and items 3 and 7 (‘I developed new interests’ and ‘I established a new path for my life’). These pairs of items were deemed to be thematically related given previously reported links between these concepts in the literature (Hefferon et al., 2009); therefore, modifications were added and the model illustrated an adequate fit; χ^2^(175) = 504.99, *p* < 0.001, Q = 2.89, CFI = 0.93, TLI = 0.91, RMSEA = 0.089 (95% CI 0.080–0.098), AIC = 616.99, SRMR = 0.05. The chi-square difference test indicated that model specifications significantly improved model fit; χ^2^(4) = 161.88, *p* < 0.001.

#### Physical Post Traumatic Growth Scale (P-PTGI; Walsh and Groarke, unpublished)

The P-PTGI (see **Supplementary Data Sheet S1**) did not demonstrate a good fit with the data: χ^2^(164) = 512.42, *p* < 0.001; *Q* = 3.21; RMSEA = 0.09 (90% CI: 0.09–0.10); CFI = 0.88; and AIC = 604.42. Five co-variances were added to the model based on recommendations from modification indices. These additional co-variances were observed to be thematically related; therefore, their inclusion was justified. Each covariance contributed significantly and improved the model which then demonstrated a very good fit to the data; χ^2^(159) = 340.19, *p* < 0.001, *Q* = 2.14, RMSEA = 0.07 (90% CI: 0.06–0.08), CFI = 0.94, AIC = 442.19, SRMR = 0.05.

#### Connor–Davidson Resilience Scale (CD-RISC; Connor and Davidson, 2003)

The CD-RISC demonstrated a poor fit: χ^2^(35) = 108.83, *p* < 0.001, *Q* = 3.11, CFI = 0.95, TLI = 0.93, RMSEA = 0.094 (95% CI 0.074–0.114), AIC = 148.83, SRMR = 0.04. Modification indices suggested that items 6 and 7 (‘able to achieve goals despite obstacles’ and ‘can stay focused under pressure’) and items 2 and 7 (‘can deal with whatever comes’ and ‘can stay focused under pressure’) should be co-varied. These pairs of items were deemed to be thematically related given previously reported links between these concepts within the PTG literature (Kashdan and Rottenberg, 2010); therefore, the modifications were added and the model improved considerably: χ^2^(33) = 72.80, *p* < 0.001, *Q* = 2.21, CFI = 0.97, TLI = 0.96, RMSEA = 0.071 (95% CI = 0.049–0.093), AIC = 116.80, SRMR = 0.03.

#### Private Body Consciousness Subscale (PBCS) of the Body Consciousness Questionnaire (BCQ; Miller et al., 1981)

The model was deemed to be an adequate fit; χ^2^(5) = 17.55, *p* = 0.004, *Q* = 3.51, CFI = 0.95, TLI = 0.91, RMSEA = 0.102 (95% CI 0.053–0.156), AIC = 37.55, SRMR = 0.04. Although RMSEA levels are sub-optimal, this measure was viewed as appropriate due to consideration of the other fit indices and the fact that no modifications were suggested.

#### Hospital and Anxiety Depression Scale (HADS; Zigmond and Snaith, 1983)

Given previous research findings illustrating weaknesses in the two-factor model of the HADS, both one- and two-factor models were subjected to CFA. The one-factor model illustrated a poor fit; χ^2^(77) = 309.21, *Q* = 4.02, CFI = 0.80, TLI = 0.77, RMSEA = 0.112 (95% CI 0.099–0.125), AIC = 365.21, SRMR = 0.08. The two-factor HADS measure also illustrated a poor fit; χ^2^(76) = 207.23, *Q* = 2.73, CFI = 0.89, TLI = 0.87, RMSEA = 0.085 (95% CI 0.071–0.099), AIC = 265.23, SRMR = 0.06, but was significantly better than the one-factor model in this population (*p* < 0.001). Therefore, modifications were conducted on the two-factor model. Modification indices suggested that item 7 (‘I can sit at ease and feel relaxed’) is problematic as it cross loads onto multiple items (item 6; ‘I feel cheerful,’ MI [24.71]). Therefore, this item was removed. In addition, a covariance between items 2 and 12 (‘I still enjoy the things I used to enjoy’ and ‘I look forward with enjoyment to things’) was suggested. These modifications were made and the resultant two-factor model (i.e., anxiety and depression) was deemed to possess good fit; χ^2^(63) = 114.22, *p* < 0.001, *Q* = 1.81, CFI = 0.95, TLI = 0.94, RMSEA = 0.058 (95% CI 0.041–0.075). AIC = 170.22, SRMR = 0.05.

### EFA Tests of Dimensionality

#### Freiburg Mindfulness Inventory (FMI; Walach et al., 2006)

First, the FMI was checked to see if it was suitable for EFA. The Kaiser–Meyer–Olkin (KMO) measure of sample adequacy was 0.92 and Bartlett’s test of sphericity was statistically significant, χ^2^(91) = 1536.59, *p* < 0.001. Levels of skewness and kurtosis were acceptable. The scree plot and parallel analysis (O’Connor, 2000) indicated a unidimensional construct (eigenvalues for actual data = 6.41, 1.27 versus parallel analysis output of 1.43, 1.32, respectively). Therefore, a one factor solution was tested. Item 13 ‘I am impatient with myself and with others’ did not meet the factor loading threshold (factor loading = 0.18) and, therefore, was removed. The eigenvalue for the retained factor was 6.38, with item factor loadings ranging from 0.56 to 0.81. Overall, the variance explained was 49.05%.

#### Patient-Oriented Prostate Utility Scale (PORPUS; Ritvo et al., 2005)

Diagnostics suggested the data were suitable for EFA [i.e., KMO = 0.74, Bartlett’s test of sphericity = χ^2^(45) = 560.44, *p* < 0.001, skewness and kurtosis were acceptable [skewness < 3; kurtosis < 10)].

While the scree plot suggested three factors, parallel analysis indicated that two factors would be most appropriate (eigenvalues for the actual data were 3.19, 1.61, and 1.14 versus eigenvalues for the parallel analysis output 1.33, 1.23, and 1.15, respectively). Therefore, a two factor solution was forced: physical symptoms (eigenvalue = 3.19; 31.89% of variance accounted for) and emotional well-being (eigenvalue = 1.61; 16.07% of variance accounted for). Item 2 ‘energy’ was found to cross-load above acceptable thresholds (i.e., 0.32) and was deleted.

## Results

### Descriptive Statistics

Reliability coefficients (and confidence intervals), means, standard deviations and scale score ranges for the psychometric measures are presented in **Table 1**. Pearson product correlations among variables included in the current study are presented in **Table 2**. The current sample were found to score relatively low on measures of anxiety and depression (i.e., anxiety and depression < mid-point on scale), and moderate levels of mindfulness, body awareness, resilience and QoL (i.e., scores > mid-point on scales). Notably, the average scores on PTG subscales were relatively low, with the exception of the appreciation of life subscale.

**Table 1:** Descriptive statistics for SEM variables (*N* = 241).

Scale	*M*	*SD*	Cronbach’s alpha (α)	95% CI	Possible range	Attained range
P-PTGI–Health Autonomy	–0.14	0.78	0.92	0.91–0.94	–2.00–2.00	–2.00–2.00
P-PTGI–Health Awareness	0.96	0.66	0.93	0.91–0.94	–2.00–2.00	–1.90–2.00
P-PTGI–Total	0.41	0.56	0.90	0.88–0.92	–2.00–2.00	–1.50–2.00
PTG–Relating to Others	13.90	10.07	0.93	0.91–0.94	0–35	0–35
PTG–New Possibilities	7.29	6.56	0.90	0.87–0.92	0–25	0–25
PTG–Personal Strength	7.19	5.65	0.88	0.86–0.91	0–20	0–20
PTG–Spiritual Change	2.77	3.29	0.82^a^	–	0–10	0–10
PTG–Appreciation of Life	7.23	4.21	0.87	0.84–0.90	0–15	0–15
PTG-Total	38.37	26.64	0.97	0.96–0.97	0–105	0–105
Mindfulness	36.83	8.65	0.91	0.89–0.93	14–56	14–52
Body Awareness	17.01	4.08	0.77	0.72–0.81	5–25	5–25
Resilience	39.08	7.53	0.93	0.91–0.94	5–50	15–50
Anxiety	5.14	3.40	0.82	0.78–0.85	0–28	0–18
Depression	4.05	3.36	0.78	0.74–0.82	0–28	0–16
Quality of Life (PORPUS Total)	71.47	15.06	0.69	0.62–0.75	0–100	23.52–100
Physical Symptoms	2.31	0.71	0.68	0.61–0.74	1–5	1–4.17
Emotional Wellbeing	1.69	0.72	0.62	0.53–0.70	1–4.33	1–4.00

*^*a*^Subscale only has two items therefore Cronbach’s alpha was not appropriate; Pearson’s correlation coefficient was calculated.*

**Table 2:** SEM correlation table.

	Subscale	1	2	3	4	5	6	7	8	9	10	11	12	13	14	15	16
1	P-PTGI –Health Autonomy	–															
2	P-PTGI–Health Awareness	0.19**	–														
3	P-PTGI–Total	0.81**	0.73**	–													
4	PTG–Relating to Others	0.18**	0.40**	0.37***	–												
5	PTG–New Possibilities	0.19**	0.40**	0.37**	0.78**	–											
6	PTG–Personal Strength	0.32**	0.42**	0.47**	0.81**	0.83**	–										
7	PTG–Spiritual Change	0.12	0.27**	0.24**	0.64**	0.66**	0.63**	–									
8	PTG–Appreciation of Life	0.13*	0.53**	0.41**	0.73**	0.75**	0.73**	0.57**	–								
9	PTG–Total	0.22**	0.46**	0.42**	0.94**	0.92**	0.92**	0.75**	0.84**	–							
10	Mindfulness	0.18**	0.12	0.19**	0.14*	0.20**	0.18**	0.17**	0.11	0.18**	–						
11	Body Awareness	0.02	0.18**	0.12	0.11	0.12	0.14*	0.10	0.08	0.13*	0.16*	–					
12	Resilience	0.32**	0.12	0.30**	0.12	0.13*	0.17**	0.10	0.09	0.14*	0.74**	0.09	–				
13	Anxiety	–0.31**	–0.01	–0.22**	–0.04	0.01	–0.03	–0.03	–0.01	–0.02	–0.46**	0.04	–0.58**	–			
14	Depression	–0.38**	–0.06	–0.30**	0.00	0.00	–0.02	–0.01	–0.02	–0.01	–0.50**	0.05	–0.54**	0.58**	–		
15	Quality of life (PORPUS Total)	0.43**	0.05	0.34**	–0.09	–0.03	–0.02	–0.08	–0.03	–0.06	0.31**	–0.06	0.40**	–0.40**	–0.55**	–	
16	Physical Symptoms	–0.36**	–0.09	–0.30**	–0.20*	0.09	0.09	0.14*	0.08	0.15*	–0.13*	0.08	–0.20**	0.19**	0.34**	0.88**	–
17	Emotional Wellbeing	–0.31**	0.03	–0.20*	–0.13*	–0.09	–0.11	–0.06	–0.06	–0.11	–0.45**	0.01	–0.50**	0.56**	0.60**	–0.66**	0.22**

*^∗∗^p < 0.01; ^∗^p < 0.05.*

### Structural Model

**FIGURE 1:**
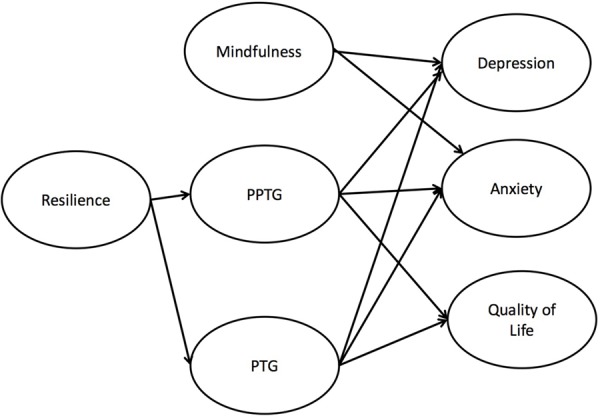
Final model testing physical post-traumatic growth as mediator between resilience and anxiety and quality of life. Post-traumatic growth did not serve as a mediator nor did physical post-traumatic growth mediate the association between resilience and depression.

A model was tested with resilience predicting PTG and PPTG, and mindfulness, PTG and PPTG predicting levels of prostate cancer quality of life, anxiety, depression, and body awareness (**Figure 1**). Correlations were included between depression and anxiety residuals as both are subscales of the HADS (e.g., Esteve et al., 2007), whilst correlations between quality of life, body awareness, depression and anxiety were deemed theoretically and empirically acceptable.

Before the structural portion of a causal model can be assessed, the validity of the measurement portion of the model must be tested (Byrne, 2009). This consists of all latent variables (e.g., PTG, PPTG, resilience, etc.) being allowed to intercorrelate freely. This model illustrated high intercorrelations between the Emotional Well-being subscale of QoL, and anxiety (*r* = 0.90) and depression (*r* = 0.89). As this indicates evidence of multi-collinearity, the emotional wellbeing subscale of QoL was removed.

The initial model depicted in **Figure 1** demonstrated a good fit; χ^2^(377) = 615.52, *p* < 0.001, *Q* = 1.63, CFI = 0.93, TLI = 0.92, RMSEA = 0.051 (95% CI = 0.042–0.058), AIC = 738.06, SRMR = 0.06. Resilience, mindfulness, PTG and PPTG accounted for 54.1% of the variance in depression, 53.6% in anxiety, 60.3% in prostate cancer physical symptom-related quality of life, and 3.8% in body awareness, with low to moderate correlations between all ‘outcome’ variables. The standardized and unstandardized regression weights for the structural equation model are shown in **Table 3**.

**Table 3:** Standardized and unstandardized regression weights for the structural equation model.

Pathway	β	*B*	*SE*	*P*
**Resilience**				
→ Physical PTG	0.49	0.15	0.04	<0.001
→ PTG	0.17	1.48	0.60	0.013
**Physical PTG**				
→ Depression	–0.50	–0.66	0.23	0.004
→ Anxiety	–0.49	–1.16	0.37	0.002
→ Quality of life	–0.86	–2.83	0.84	<0.001
→ Body awareness	0.08	0.20	0.29	0.50
**PTG**				
→ Depression	0.28	0.01	0.00	0.004
→ Anxiety	0.29	0.02	0.01	<0.001
→ Quality of life	0.53	0.06	0.01	<0.001
→ Body awareness	0.11	0.01	0.01	0.22
**Mindfulness**				
→ Depression	–0.50	–0.21	0.05	<0.001
→ Anxiety	–0.51	–0.38	0.06	<0.001
→ Quality of life	0.02	0.02	0.11	0.87
→ Body awareness	0.01	0.07	0.07	0.29

In support of the study’s hypotheses, PPTG significantly negatively predicted depression (β = -0.50, *p* = 0.004), anxiety (β = -0.49, *p* = 0.002) and quality of life symptoms (β = -0.86, *p* < 0.001). The pathways from PPTG to body awareness was non-significant (*p* > 0.05). Thus, higher PPTG predicted lower depression, lower anxiety and better quality of life.

A non-directional hypothesis was proposed to examine the relationship between PTG and distress (i.e., depression and anxiety) given previously inconsistent findings. It was found that PTG significantly positively predicted depression (β = 0.28, *p* = 0.004) and anxiety (β = 0.29, *p* < 0.001). PTG significantly predicted lower levels of quality of life (β = 0.53, *p* < 0.001). It was also hypothesized that those with higher levels of PTG would exhibit higher levels of body awareness; however, it was found that the pathway to body awareness was non-significant (*p* > 0.05). Thus, higher levels of PTG predicted higher levels of depression and anxiety, and lower levels of quality of life.

In the current model, it was hypothesized that higher levels of mindfulness would be associated with greater body awareness, quality of life and lower levels of distress (i.e., depression and anxiety). As predicted, mindfulness was found to significantly negatively predict depression (β = -0.50, *p* < 0.001) and anxiety (β = -0.51, *p* < 0.001). However, interestingly, pathways to quality of life and body awareness were statistically non-significant (*p*s > 0.05). Thus, higher levels of mindfulness predicted lower levels of depression and anxiety.

Following the testing of the hypothesized model, the outcome variable body awareness was deleted as no statistically significant relationships between this construct and the predictors were observed. This significantly improved model fit; χ^2^(49) = 0.89.46, *p* < 0.001, with final model indices showing a good fit to the data: χ^2^(328) = 526.06, *p* < 0.001, *Q* = 1.60, CFI = 0.94, TLI = 0.93, RMSEA = 0.051 (95% CI = 0.042–0.058), AIC = 738.06, SRMR = 0.06.

The current model hypothesized that the relationship between resilience and all outcome variables is mediated by PTG and PPTG. Thus, a model was conducted which stipulated direct relationships between resilience and outcomes, in addition to indirect mediated relationships through PTG and PPTG. Model fit was good: χ^2^(325) = 519.16, *p* < 0.001, *Q* = 1.60, CFI = 0.94, TLI = 0.93, RMSEA = 0.050 (95% CI = 0.042–0.058), AIC = 681.16, SRMR = 0.06. The direct pathways between resilience and outcomes (i.e., anxiety, depression, and QoL) were non-significant (*p*s < 0.05). Using IBM AMOS 23 bootstrapping, with 5,000 samples and 90% bias-corrected non-standardized confidence intervals, significant indirect pathways were observed between resilience and depression (*p =* 0.006, 90% CI = -0.55 to -0.04), resilience and anxiety (*p* = 0.028, 90% CI = -0.61 to -0.03), and resilience and QoL (*p* = 0.004, 90% CI = -2.51 to -0.21). Furthermore, a user-defined estimand was conducted in AMOS 23 following MacKinnon et al.’s (2007) and Hayes’ (2009) mediation approach, to examine which indirect pathways were significant. Two individual mediation pathways were found to be significant: (1) resilience → PPTG → QoL (unstandardized effect = -0.39, *SE* = 0.05, *p* < 0.001), and (2) resilience → PPTG → anxiety (unstandardized effect = -0.16, *SE* = 0.21, *p* = 0.01). Other indirect effects were non-significant (*p*s > 0.05).

## Discussion

A model was tested with PTG and PPTG predicting levels of prostate cancer quality of life, anxiety, depression and body awareness. The current model explored many new facets of PTG and has looked at the relationship between commonly implicated variables in the PTG process albeit in a new way. Resilience is an integral part of the current model. Resilience is a target of interest for PTG research as it has been cited as a key component which can manipulate the level of cognitive processing engaged in by an individual following a trauma (Calhoun and Tedeschi, 2006). In the current model, resilience acts together with PTG and PPTG in predicting outcomes in a variety of areas such as distress and quality of life.

### Cognitive Processing within PTG and PPTG

A mediation model was conducted and showed that resilience had a significant indirect effect on anxiety and symptom-related quality of life though PPTG. Notably, when individual indirect pathways were examined, PPTG was found to significantly mediate the relationship between resilience and anxiety, and resilience and symptom-related quality of life. Resilience was hypothesized to play a role in PTG as traumatic experiences may be less traumatic to resilient individuals (Bonanno et al., 2004). Results here suggest that greater resilience is related to greater PTG and PPTG. This model exhibited a good fit and suggests that the relationship between higher resilience and better adjustment is mediated by physical PTG. While this finding is contrary to the existing theoretical framework whereby it is suggested that higher resilience is associated with lower PTG (Tedeschi and Calhoun, 2004), the current study findings are in line with more recent research with Australian prostate cancer survivors (Wilson et al., 2014).

Importantly, what this PPTG model highlights is how the experience of physical trauma and the inclusion of the physical aspect of illness/trauma extends our understanding of adjustment in post trauma and is a key incremental advance for PTG research. This model extends the previous theoretical framework of Tedeschi and Calhoun (1996) by considering the role of physical trauma and the body within the growth experience especially in relation to quality of life and distress. The role of PPTG in adjustment is a crucial finding in terms of the future conceptualization of PTG. Interestingly, the current model results may only be counter to the predominant theoretical framework (i.e., results here suggests that greater resilience is related to greater PTG and PPTG which is opposite to existing theories), because PPTG does not center on cognitive processing as much as other dimensions of growth. This could be due to the corporeal nature of physical trauma and the subsequent physical recovery and rejuvenation experienced (Hefferon, 2012). Future research could usefully assess how resilience and other predetermining factors of PTG and PPTG impact and potentially mediate the outcome variables used in the current study. This may shed further light on the mechanisms which drive positive growth. Importantly, this model suggests that resilience is mediated by PPTG, as opposed to PTG. This is a key point for future research as this is the first study to explore resilience and growth in this way.

It is proposed that previous inconsistent findings on the relationship between growth and distress (Helgeson et al., 2006) may be a direct result of not differentiating between internal and external transgressors (Hefferon, 2012). Given the adaptive role that PPTG appears to play in distress within this population, there are some other issues which may be useful to explore in terms of the unique impact of a *physical* trauma on distress. For instance, previously, Morris and Shakespeare-Finch (2011) found that trauma severity was directly related to distress, but not to traditional PTG. As there was formerly no acknowledgment of the physical self within any PTG measure (Park and Lechner, 2006), it is feasible that these previous findings do not adequately capture the entirety of the relationship between distress, PTG and trauma severity. As Hefferon (2012) has asserted a “new addition of a more embodied perception of PTG, which dictates that as embodied individuals, any trauma caused unto or within the body will entail a different reconstruction and journey to PTG” (p. 1241).

### Adjustment within the PTG and PPTG

As past findings have been inconsistent in terms of quality of life, the inclusion of PPTG is an important addition to the model of growth. Interestingly, higher PPTG results in greater quality of life for prostate cancer survivors explaining 67.24% of the variance. PTG, as traditionally defined, has often predicted poorer quality of life in previous studies (Mohr et al., 1999; Tomich and Helgeson, 2004). This is contrary to expectation given that PTG has been previously conceptualized to improve well-being (Tedeschi and Calhoun, 2004). Some research has suggested that the low levels of quality of life may have been associated with ongoing engagement with distress and rumination and that growth outcomes could be linked to this deliberate rumination which, in turn, may affect quality of life (Stockton et al., 2011).

The current model suggests that, perhaps, it is not *just* the continuation of the in-depth cognitive processing which influences the relationship between PTG and quality of life. Perhaps it is the absence of any aspect or acknowledgment of the role of the physical self in well-being and adjustment following trauma. Thus, by including the physical dimension, findings might elucidate the adjustment process further, while without the physical dimension, traditional PTG may continue to appear to have an inconsistent relationship with quality of life.

### Implications for the Conceptualization of PTG

This model suggests that PPTG is a better predictor of decreased levels of anxiety, depression and quality of life when compared to PTG due to higher standardized regression weights within the model. This is interesting as, perhaps, the more embodied perspective of PPTG predicts better outcomes for survivors following a physical trauma such as illness. It is clear from the current model that both conceptualizations of PTG are valuable and that all six dimensions have a contribution to make in terms of survivorship and well-being.

It is also important to look at strong and weak associations within any structural equation model. There is a particularly strong association between quality of life and PPTG, while there is a much smaller negative relationship between traditional PTG and quality of life. The negative correlation between PTG and quality of life has been previously discussed by Thornton and Perez (2006), in the context of how the distress may act as a catalyst in the formation of PTG and that the adaptive significance of PTG in terms of results may be confounded by ongoing cancer-related distress.

A lack of relationship between body awareness and any of the variables was unexpected; however, this could be related to the sample being one of older adult males. Males have been noted, as a group, to have lower levels of body awareness (Shields et al., 1989). The impact of masculinity on body awareness is a point to consider in relation to the future development and improvement of the concept of PPTG. This study also hypothesized that higher levels of mindfulness would be associated with quality of life. Although mindfulness was found to significantly negatively predict depression and anxiety, pathways to quality of life were statistically non-significant. However, as reported in the results section, it must be acknowledged that the measure of quality of life as used in the current study focuses on physical symptoms related to quality of life. This may therefore have impacted the predicted relationships between mindfulness and quality of life. Further research with other quality of life measures and different cohorts is required.

It is important to note, given the early stage of theory development, that PPTG, as it is currently conceptualized in this study, may not represent *all* experiences of PPTG. Future research should consider other variables which may impact on the PPTG experience (e.g., trauma severity).

### Strengths and Limitations

One of the key strengths of this research is that it addresses a significant gap in PTG research by incorporating aspects of growth following *physical* trauma. This has been cited as a need in the PTG field for over a decade (Park and Lechner, 2006). This research highlights the importance of the embodied approach to PTG following physical illness (Hefferon, 2012).

There are some limitations to the current research. It is acknowledged that the process of growth is an iterative one (Leal et al., 2014). Therefore, future research would benefit from the inclusion of longitudinal data, which would enable a deeper understanding of the temporal relationship between both aspects of PTG and the outcome variables.

Participants were not only recruited from local clinics and local support groups, but also via popular prostate cancer forums. Therefore, some of the participants may differ from the general population of survivors as they were already seeking support from a prostate cancer support group. Also, due to the nature of the study design (i.e., cross sectional design), it is not possible to fully examine reciprocal relationships between study variables within the post traumatic process (e.g., distress could be a predictor of PTG/PPTG and not only an outcome variable). Future research with longitudinal designs is needed to further explore these associations. In addition, it is important to investigate pertinent variables that may impact adjustment specific to this cohort (i.e., marital status, trauma severity and fear of reoccurrence). Further, in terms of measurement issues, due to the presence of a strong general factor, the HADS does not provide good separation between symptoms of anxiety and depression, and consequently is best used as a measure of general distress (Norton et al., 2013).

## Conclusion

Findings suggest the central role of PTG in the prostate cancer survivorship experience is enhanced by the inclusion of PPTG. Adjusting to a physical trauma such as illness (internal transgressor) is unlike a trauma with an external transgressor as the physical trauma creates an entirely different framework for adjustment. The current study demonstrates the impact of PPTG on adjustment. This significantly adds to the theory of the development of PTG by highlighting the interplay of resilience with PTG, PPTG and adjustment. The development of the PPTG construct presents a more comprehensive picture of post traumatic reactions, particularly in terms of the male experience of PPTG following prostate cancer. These findings lay the foundation for potential development of interventions to improve adjustment of older males following prostate cancer.

## Ethics Statement

This study was carried out in accordance with the recommendations of the Helsinki declaration and the guidelines of the International Committee of Medical Journal Editors. All subjects gave written informed consent in accordance with the Declaration of Helsinki. The protocol was approved by the NUI Galway Research Ethics Committee and the University Hospital Galway Research Ethics Committee.

## Author Contributions

DW collected and analyzed the data. DW and AG conceptualized the study. DW led on the manuscript writing. AG, TM, and RC provided feedback on drafts of the manuscript. All authors approved the final manuscript draft.

## Conflict of Interest Statement

The authors declare that the research was conducted in the absence of any commercial or financial relationships that could be construed as a potential conflict of interest. The reviewer SC, and handling Editor declared their shared affiliation.

## Supplementary Material

DATA SHEET S1 Physical Post Traumatic Growth Inventory [P-PTGI].
