# Supplementary material for: A Model to Predict Psychological- and Health-Related Adjustment in Men with Prostate Cancer: The Role of Post Traumatic Growth, Physical Post Traumatic Growth, Resilience and Mindfulness
Source: Front Psychol. 2018 Feb 15;9:136. doi: 10.3389/fpsyg.2018.00136 (PMC5818687; doi:10.3389/fpsyg.2018.00136)
Supplement: DATA SHEET S1 — Physical Post Traumatic Growth Inventory [P-PTGI]. [file Data_Sheet_1.DOCX]

Please read each statement considering how you feel now after your diagnosis and treatment. Please tick the box which most applies to you.

|  | Greatly decreased | Somewhat decreased | Not changed | Somewhat increased | Greatly increased | Not applicable to me |
| --- | --- | --- | --- | --- | --- | --- |
| My trust in my body has… |  |  |  |  |  |  |
| The feeling that I have control over my health has… |  |  |  |  |  |  |
| My confidence in my body has… |  |  |  |  |  |  |
| The empowerment I feel physically has… |  |  |  |  |  |  |
| The control I feel over my body has… |  |  |  |  |  |  |
| The feeling that I have overcome any negative physical changes has… |  |  |  |  |  |  |
| I feel the physical strength in my body has… |  |  |  |  |  |  |
| My confidence that my body will be strong enough to recover has… |  |  |  |  |  |  |
| The feeling that my body is reliable has… |  |  |  |  |  |  |
| The sense of achievement in overcoming the physical obstacles of my illness has… |  |  |  |  |  |  |
| My awareness of my own body has… |  |  |  |  |  |  |
| The amount I listen to my body has… |  |  |  |  |  |  |
| The amount I monitor my body has… |  |  |  |  |  |  |
| My concern for my overall health has… |  |  |  |  |  |  |
| My responsibility for my health has… |  |  |  |  |  |  |
| The attention I pay to how my body works has… |  |  |  |  |  |  |
| My awareness of parts of my body has… |  |  |  |  |  |  |
| The care I give my body has… |  |  |  |  |  |  |
| My appreciation for good health has… |  |  |  |  |  |  |
| The time I put into researching information about my health has… |  |  |  |  |  |  |
| My trust in my body has… |  |  |  |  |  |  |
